# Nudging epidemic policy compliance: experimental insights into message framing

**DOI:** 10.3389/fpubh.2025.1587987

**Published:** 2025-07-23

**Authors:** Biao Xu, Patiman Yidilisi, Hailing Xi, Shuyan Gu

**Affiliations:** School of Government, Nanjing University, Nanjing, China

**Keywords:** public compliance, health policy, framing effect, behavioral public policy, nudging

## Abstract

**Objective:**

Achieving widespread voluntary public compliance is critical for effective epidemic management. This study investigates how different message-framing strategies influence individuals’ willingness to comply with public health measures during a simulated epidemic scenario.

**Methods:**

Using a randomized 2 × 2 experimental design, we tested the relative effectiveness of four framing conditions—gain-private, loss-private, gain-social, and loss-social—on compliance intentions. Participants (*N* = 391) were randomly assigned to one of these conditions or a no-framing control group. Compliance willingness was assessed through self-reported intentions to adhere to recommended preventive behaviors.

**Results:**

Framed messages significantly increased compliance intentions compared to the control condition. Among framing strategies, the loss-social frame (emphasizing negative societal consequences of noncompliance) demonstrated the strongest effect, followed by gain-private, gain-social, and loss-private frames. Pairwise comparisons revealed important interactions: gain-framing was more effective within private motivational contexts, whereas loss-framing was particularly compelling within social contexts. Critically, loss-social messages were significantly superior to loss-private ones, while gain-social and gain-private messages performed similarly.

**Conclusion:**

Strategic message framing effectively enhances public compliance during epidemic crises, with loss-social framing emerging as the most potent approach. These findings offer critical insights for policymakers and health communicators, recommending targeted use of loss-social messaging to optimize public adherence to epidemic prevention guidelines.

## 1 Introduction

Infectious diseases have emerged as critical threats in the globalized, interconnected twenty-first century, challenging health systems worldwide. Epidemic outbreaks such as SARS, Ebola, and particularly the COVID-19 pandemic underscore the essential role of public health policies in containing infectious diseases through coordinated behavioral interventions (1–3). Despite theoretical foundations and scientific backing, the effectiveness of these policies fundamentally hinges on achieving widespread voluntary public compliance—a goal frequently undermined by behavioral inertia and motivational barriers (4–6). Hence, enhancing compliance behavior is critical for successful epidemic management.

Traditional approaches to improving compliance typically emphasize educational interventions and incentive-based systems. Educational campaigns increase awareness and knowledge of disease risks and preventive behaviors, while incentives—such as rewards or penalties—motivate individuals through rational cost–benefit analyses (7, 8). However, both strategies are fundamentally grounded in rational choice theory, which assumes that individuals make decisions based on logical evaluation of benefits and costs. During public health crises, these rationally oriented strategies have consistently faced limitations. Factors such as cognitive overload, perceived invulnerability, habitual noncompliance, and psychological fatigue diminish the effectiveness of purely rational appeals (9–11). Moreover, both educational and incentive-based interventions often demand significant resources and preparation time, making them impractical for rapid deployment in urgent epidemic scenarios (12, 13).

Recognizing these limitations, recent studies in behavioral economics and psychology highlights the potential of leveraging automatic, heuristic-driven decision processes—termed System 1—as opposed to slower, analytical reasoning processes (System 2), for promoting compliance in emergency contexts (14). Given that preventive health behaviors such as mask-wearing, social distancing, and hand hygiene are typically repetitive and habitual, interventions targeting intuitive decision-making offer considerable promise (15). One particularly effective behavioral strategy emerging from this literature is the use of message framing, which exploits cognitive biases in human decision-making described by prospect theory (16).

More broadly, framing effects describe how the strategic presentation of choices or information can significantly influence behavioral decisions, whether by emphasizing potential gains versus losses (17) or by directing focus through the way actions themselves are described (18). However, much of this literature centers on individual (private) benefits or risks, neglecting the inherently social nature of epidemic prevention measures. Compliance with epidemic policies frequently yields collective benefits such as reducing disease transmission, protecting vulnerable populations, and relieving healthcare system burdens—benefits that private-focused framing might insufficiently capture. Therefore, extending existing research, this study systematically investigates how the interaction between goal framing (gain vs. loss) and motivational framing (private vs. social) influences individuals’ willingness to adhere to epidemic control measures during a simulated public health crisis. Using a rigorous experimental design, we explore the differential effectiveness of four distinct framing strategies—gain-private, loss-private, gain-social, and loss-social—in promoting voluntary compliance behaviors.

Theoretically, this study advances framing-effect research by integrating private and social motivational dimensions into the gain-loss framing paradigm, providing nuanced insights into behavioral decision-making under uncertainty and high-stakes conditions. Practically, the findings offer policymakers actionable, cost-effective communication strategies for rapidly enhancing public compliance during epidemics. Beyond immediate public health applications, this research provides valuable guidance for addressing broader global challenges requiring coordinated individual and collective action, such as climate change mitigation and urban health management.

## 2 Hypotheses

### 2.1 Goal framing: gain versus loss

According to goal-framing theory, how information is presented—either emphasizing potential benefits (gain-framing) or highlighting potential costs (loss-framing)—significantly shapes behavioral outcomes by affecting individuals’ risk perceptions and motivations (17, 19). Typically, gain-framed messages, which stress positive outcomes of compliance, are shown to be effective in promoting preventive health behaviors, as they align with people’s general preference for securing certain, beneficial outcomes (17, 20). Conversely, loss-framed messages, emphasizing potential negative consequences of non-compliance, are often persuasive in contexts involving higher perceived risks, as they activate people’s aversion to losses (16).

However, recent empirical studies in health contexts—such as COVID-19 vaccination—have shown that loss-framed messages can sometimes outperform gain-framed messages due to heightened public sensitivity to risks and uncertainties inherent in novel health crises (21, 22). Given these mixed findings and the context-dependent nature of framing effects, we hypothesize:

*H1*: Framed messages significantly increase individuals' willingness to comply with epidemic control measures compared to non-framed messages.

*H2*: Gain-framed messages are generally more effective in promoting compliance behaviors than loss-framed messages, given the preventive nature of epidemic control measures.

### 2.2 Motivational framing: private versus social

Beyond gain-loss framing, motivational framing emphasizes the beneficiary of compliance—either oneself (private framing) or others and society at large (social framing). Private-framed messages focus on individual-level outcomes, such as personal health and economic stability, relying primarily on egoistic motivations. Social-framed messages, in contrast, highlight collective-level outcomes such as reducing transmission, protecting vulnerable populations, and easing burdens on healthcare systems, thereby appealing to altruism and social responsibility (23–25).

Research indicates that social framing may better address compliance behaviors involving positive externalities, such as epidemic control measures, by mitigating free-rider problems and activating collective motivations (21). However, existing literature remains inconclusive regarding whether private or social framing is inherently more effective, especially when intersecting with gain-loss framing. Thus, we propose:

*H3*: Social-framed messages have a significantly stronger impact on compliance intentions than private-framed messages, due to their explicit emphasis on collective benefits and externalities.

### 2.3 Interaction effects: combining goal and motivational frames

Understanding the nuanced interactions between goal framing and motivational framing is crucial. Specifically, we anticipate differential effects when combining these two framing strategies. For instance, private motivations may align more intuitively with gain framing due to the direct personal benefits emphasized, whereas social motivations might be more compelling under loss framing, as it accentuates the negative societal consequences of non-compliance. Hence, we further hypothesize:

*H4a*: Within private motivational framing, gain-framed messages will lead to greater willingness to comply than loss-framed messages.

*H4b*: Within social motivational framing, loss-framed messages will be more effective than gain-framed messages due to heightened sensitivity to societal risks.

Within a gain-framed context, adding a social-benefit emphasis can amplify compliance motivation by invoking prosocial rewards and norms beyond mere self-interest. Behavioral theory on “warm-glow” giving suggests that people experience an inherent emotional reward from contributing to the welfare of others (26). Empirical evidence supports this: health messages highlighting benefits to others have outperformed self-focused appeals in promoting preventive behaviors. For example, research found that hospital hand-hygiene compliance rose when staff were reminded that hand-washing protects patients rather than just themselves, leveraging caregivers’ altruistic motives (27). We therefore expect that aligning gain-framed messaging with social (rather than private) motivators will more strongly encourage voluntary compliance:

*H5a*: Within gain-framed contexts, social motivational framing will elicit significantly stronger compliance intentions than private motivational framing.

Conversely, within loss-framed contexts—characterized by highlighting negative consequences of non-compliance—motivational framing may significantly alter the message’s effectiveness. The literature suggests that highlighting losses to society may be particularly compelling because individuals tend to feel stronger moral obligations and greater psychological distress regarding harms to their community rather than to themselves alone. Therefore, emphasizing the broader societal impacts of noncompliance (such as increased disease transmission, overwhelmed healthcare systems, or societal instability) might elicit stronger emotional and behavioral responses than emphasizing personal harms, such as individual health risks or economic disruptions. Thus, our final hypothesis is:

*H5b*: Within loss-framed contexts, social motivational framing will result in significantly stronger compliance intentions compared to private motivational framing, due to heightened emotional and moral responsiveness to potential collective losses.

By explicitly testing these hypotheses, this study provides clarity on how specific combinations of framing strategies influence compliance behavior, thereby refining theoretical frameworks and enhancing practical applications for policy communication.

## 3 Methods

### 3.1 Context and scenario selection

The context selected for this study is a hypothetical resurgence scenario of COVID-19 following the initial wave. This scenario was particularly suitable as it realistically captures public health concerns under uncertainty, high stakes, and immediate behavioral demands. Participants imagined a situation where a highly infectious and hazardous new COVID-19 variant emerged after the initial outbreak, resulting in no available vaccines or effective antiviral treatments. Consequently, public authorities reintroduced comprehensive non-pharmaceutical interventions (NPIs), including mandatory mask-wearing, strict social distancing protocols, and travel restrictions. This scenario mirrors actual public health emergencies, thereby providing ecological validity for testing compliance behaviors.

### 3.2 Experimental design

A randomized between-subjects experimental design was implemented using the Credamo online survey platform between April 10 and May 15, 2023. Participants (initial *N* = 413) were randomly assigned with equal probability into one of five experimental conditions: (1) Gain-Private, (2) Loss-Private, (3) Gain-Social, (4) Loss-Social, and (5) Control (no framing message). Randomization was achieved through Credamo’s built-in randomizer, ensuring allocation concealment from both participants and researchers, thus safeguarding internal validity.

Initially, all participants read an identical baseline scenario about the emerging infectious disease (Appendix Table A). Subsequently, participants in each experimental condition received specific framing messages:

Gain-private frame: Emphasized personal benefits of compliance, including reducing individual infection risk, maintaining good health, and ensuring stable daily life and income.

Loss-private frame: Highlighted personal risks of non-compliance, including illness, severe symptoms, potential life-threatening outcomes, disrupted daily routines, and economic instability.

Gain-social frame: Stressed collective benefits of compliance, such as reducing disease transmission, protecting vulnerable populations (older adults and children), alleviating healthcare system pressure, and accelerating economic and social recovery.

Loss-social frame: Underscored societal risks of non-compliance, including increasing infection risks to vulnerable groups, worsening epidemic spread, burdening healthcare systems, and delaying social and economic recovery.

Control group: Received no specific framed message, serving as a baseline to assess spontaneous willingness to comply without external framing influence.

Following message exposure, participants reported their willingness to comply with three specific public health measures: movement restrictions, personal protective behaviors, and health monitoring.

### 3.3 Participants and procedures

**Figure 1:**
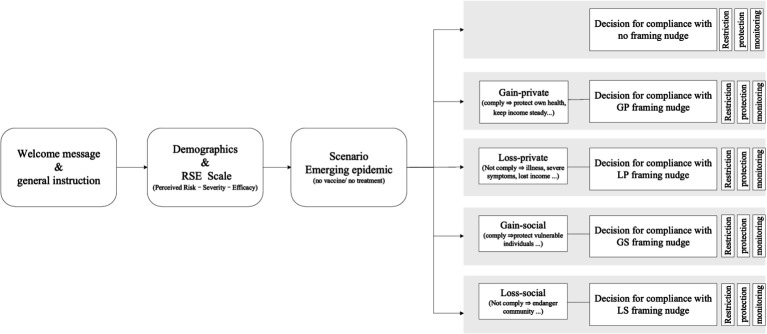
Flow of participants and framing stimuli across experimental arms.

Participants were recruited through three distinct channels to enhance sample diversity and representativeness: (i) university announcements offering monetary incentives (¥5) or academic credits; (ii) targeted advertisements via Credamo to meet national demographic quotas; and (iii) snowball sampling leveraging the authors’ social and professional networks. Electronic informed consent was obtained prior to participation, and the online survey was administered via Credamo’s secure platform. Participant flow and procedures are detailed in Figure 1.

Data quality assurance included two instructional manipulation checks embedded within the survey to verify participant attention. Of the initial 413 participants recruited, 22 were excluded for failing these checks, leaving a final analytical sample of 391 participants. Our largest regression model includes 12 predictors. Conventional rules of thumb recommend 10–20 observations for each predictor (28). With 391 cases we exceed the upper bound (20 × 12 = 240), reducing the risk of over-fitting and unstable coefficients.

### 3.4 Measures

The primary dependent variable, willingness to comply, was assessed using a validated 5-point Likert scale, with response options ranging from 1 = “Not at all willing” to 5 = “Extremely willing.” The specific question posed to participants was: “Are you willing to comply with these epidemic policies?,” and the question wordings were taken and adapted from previous research (21). Additionally, participants reported socio-demographic information including age, gender, occupation, education level, income, and changes in income.

This study also investigated several variables that could influence compliance willingness, consistent with findings in the literature. The three of the questions used to assess these factors were also adapted from Gong et al. (21). Specifically, the perceived probability of future infection with a high-risk emergent infectious disease was measured by the question: “If there were a second wave of COVID-19 now, would I think it highly likely that I would be infected?,” with choices ranging from “strongly disagree” (1) to “strongly agree” (5). Participants were then asked about the perceived severity of the pandemic with the question: “If there were a second wave of COVID-19 now, I think it would still be a serious pandemic,” and the responses ranged from “totally disagree (1)” to “strongly agree (5).” Finally, the perceived effectiveness of complying with preventive behaviors policies was evaluated by the following question: “If a second wave of COVID-19 were to occur now, following preventive behaviors policies would be effective in preventing me from becoming infected,” on a scale of 1 (strongly disagree) to 5 (strongly agree). Furthermore, we incorporate two additional variables that may influence individuals’ compliance willingness: the capacity of their workplace (the maximum number of people a workplace can accommodate) and the presence of vulnerable family members who are susceptible to the pandemic.

### 3.5 Statistical analysis

Ordinal logistic regression (OLR) was employed to analyze data due to the ordinal nature of the outcome variable, willingness to comply. OLR is appropriate for modeling ordered categorical data, ensuring robust estimates and facilitating meaningful interpretation of cumulative compliance likelihood. Prior to the main analysis, the proportional odds assumption, essential for OLR validity, was rigorously evaluated using the Brant test. The omnibus Brant test confirmed assumption satisfaction (*p* = 0.104), thus validating the chosen analytical method.

Both unadjusted and adjusted regression models were computed to quantify the direct effects of different framing strategies. Adjusted models included socio-demographic covariates, perceived risk and severity, and perceived compliance efficacy measures to rigorously isolate framing effects. Due to the 2×2 factorial design, which combined goal framing (gain/loss) and motivational framing (private/social), hypotheses regarding the main effects of gain/loss (H2) and private/social (H3) framing were primarily examined through pairwise comparisons within the relevant framing dimensions. Specifically, the general prediction in H3 regarding the superiority of social framing was tested by comparing Gain-Private to Gain-Social messages and Loss-Private to Loss-Social messages. Pairwise comparisons among all experimental conditions were conducted via Wald tests to determine statistically significant differences in effectiveness between the frames. Statistical significance thresholds were consistently set at *p* < 0.05. All analyses were performed using Stata/MP 16.0 (Stata Corp, College Station, TX).

## 4 Results

**Figure 2:**
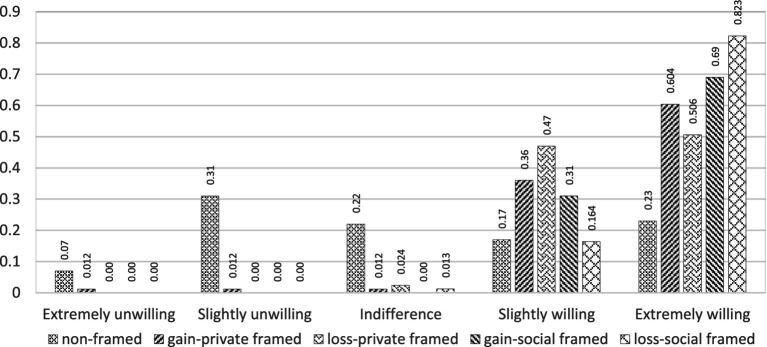
Willingness to comply in different framing.

A total of 391 participants were randomly assigned across five experimental conditions: Gain-private (*n* = 81), Loss-private (*n* = 81), Gain-social (*n* = 79), Loss-social (*n* = 79), and No-framing Control (*n* = 71). Demographic characteristics, including age, gender, education, health status, and income, are detailed in Table 1. There were no statistically significant differences in these characteristics across groups, confirming effective randomization. Figure 2 illustrates the distribution of participants’ compliance intentions across conditions. Notably, participants exposed to framed messages reported higher willingness to comply compared to the control group. Specifically, the proportion reporting “extreme willingness” was highest in the Loss-social condition (82.3%), followed by Gain-social (69.0%), Gain-private (60.4%), and Loss-private (50.6%) conditions. In contrast, only 23.0% of control group participants expressed “extreme willingness,” indicating a substantial framing effect.

**Table 1:** Sample characteristics and their differences in experimental conditions.

Variables	Total (*N* = 391)	Non-framed (*N* = 71)	Gain-private framed (*N* = 81)	Loss-private framed (*N* = 81)	Gain-social framed (*N* = 79)	Loss-social framed (*N* = 79)	*p* value
Gender^2^							
Female	269 (68.80)	52 (73.24)	56 (69.14)	57 (70.37)	50 (63.29)	54 (68.35)	*χ*^2^ = 1.87 *p* = 0.76
Male	122 (31.20)	19 (26.76)	25 (30.86)	24 (29.63)	29 (36.71)	25 (31.65)	
Health^4^							
Poor health	7 (1.79)	4 (5.63)	0 (0.00)	3 (3.70)	0 (0.00)	0 (0.00)	*χ*^2^ = 20.66 *p* = 0.06
Fair	77 (19.69)	15 (21.13)	12 (14.81)	18 (22.22)	13 (16.46)	19 (24.05)	
Good	304 (77.75)	51 (71.83)	67 (82.72)	60 (74.07)	66 (83.54)	60 (75.95)	
Unable to assess	3 (0.77)	1 (1.41)	2 (1.41)	0 (0.00)	0 (0.00)	0 (0.00)	
Occupation^5^							
Student	440(87.13)	5(100)	37(94.87)	24(85.71)	205(90.71)	169(81.64)	*χ*^2^ = 38.22 *p* = 0.001
Government institution	15(2.97)	0(0.00)	0(0.00)	2(7.14)	7(3.10)	6(2.90)	
National administrative agency	40(7.92)	0(0.00)	1(2.56)	0(0.00)	12(5.31)	27(13.04)	
State-owned enterprise	5(0.99)	0(0.00)	1(2.56)	2(7.14)	2(0.88)	0(0.00)	
Private enterprise	5(0.99)	0(0.00)	0(0.00)	0(0.00)	0(0.00)	5(2.42)	
Education^5^							
Senior High School	16 (4.09)	3 (4.23)	3 (3.70)	6 (7.41)	2 (2.53)	2 (2.53)	*χ*^2^ = 24.95 *P* = 0.07
Associate degree	10 (2.56)	3 (4.23)	1 (1.23)	3 (3.70)	3 (3.80)	0 (0.00)	
Bachelor’s Degree	272 (69.57)	38 (53.52)	57 (70.37)	62 (76.54)	54 (68.35)	61 (77.22)	
Master’s Degree	86 (21.99)	25 (35.21)	19 (23.46)	8 (9.88)	18 (22.78)	16 (20.25)	
Ph.D.	7 (1.79)	2 (2.82)	1 (1.23)	2 (2.47)	2 (2.53)	0 (0.00)	
Cross-regional work^2^							
No	115(22.77)	1(20.00)	7(17.95)	3(10.71)	43(19.03)	61(29.47)	*χ*^2^ = 9.93 *p* = 0.042
Yes	390(77.23)	4(80.00)	32(82.05)	25(89.29)	183(80.97)	146(70.53)	
Vulnerable family members^2^							
No	180 (46.04)	40 (56.34)	39 (48.15)	36 (44.44)	32 (40.51)	33 (41.77)	*χ*^2^ = 4.81 *p* = 0.31
Yes	211 (53.96)	31 (56.34)	42 (51.85)	45 (55.56)	47 (59.49)	46 (58.23)	
Family annual income^6^							
<= 10 K	6 (1.53)	0 (0.00)	4 (4.94)	0 (0.00)	0 (0.00)	2 (2.53)	*χ*^2^ = 29.93 *p* = 0.19
10-30 K	35 (8.95)	9 (12.68)	6 (7.41)	8 (9.88)	5 (6.33)	7 (8.86)	
30-80 K	54 (13.81)	14 (19.72)	10 (12.35)	11 (13.58)	10 (12.66)	9 (11.39)	
80-150 K	102 (26.09)	14 (19.72)	25 (30.86)	17 (20.99)	19 (24.05)	27 (34.18)	
150-300 K	133 (34.02)	23 (32.39)	28 (34.57)	28 (34.57)	33 (41.77)	21 (26.58)	
300-1000 K	60 (15.35)	11 (15.49)	7 (8.64)	17 (20.99)	12 (15.19)	13 (16.46)	
> = 1,000 K	1 (0.26)	0 (0.00)	1 (1.23)	0 (0.00)	0 (0.00)	0 (0.00)	
Income change^3^							
Increased	95 (24.30)	13 (18.31)	19 (23.46)	19 (23.46)	21 (26.58)	23 (29.11)	*χ*^2^ = 7.48 *p* = 0.49
Decreased	109 (27.88)	23 (32.39)	27 (33.33)	17 (20.99)	24 (30.38)	18 (22.78)	
Unchanged	187 (47.83)	35 (49.30)	35 (43.21)	45 (55.56)	34 (43.04)	38 (48.10)	
Age	25.74(8.13)	23.34(5.90)	24.53(6.83)	26.76(10.99)	27.66(7.77)	26.16(7.47)	*F* = 1.10 *p* = 0.33
Capacity	26.27(16.52)	29.08(16.92)	27.06(16.25)	22.33(17.27)	25.06(16.09)	28.25(15.59)	*F* = 0.85 *p* = 0.75
Perceived probability of infection^5^							
Strongly disagree	10(2.56)	2(2.28)	3(3.70)	2(2.47)	1(1.27)	2(2.53)	*F = 3.46, p = 0.0086*
Somewhat disagree	76(19.44)	13(18.31)	20(24.69)	11(13.58)	13(16.46)	19(24.05)	
Neither agree nor disagree	96(24.55)	27(38.03)	20(24.69)	22(27.16)	13(16.46)	14(17.72)	
Somewhat agree	152(38.87)	25(35.21)	30(37.04)	29(35.80)	39(49.37)	29(36.71)	
Strongly agree	57(14.58)	4(5.63)	8(9.88)	17(20.99)	13(16.46)	15(18.99)	
Perceived severity of pandemic^5^							
Strongly disagree	14(3.58)	4(5.63)	3(3.70)	1(1.23)	2(2.53)	4(5.06)	*F = 1.51, p = 0.1971*
Somewhat disagree	106(27.11)	23(32.39)	23(28.40)	22(27.16)	17(21.52)	21(26.58)	
Neither agree nor disagree	83(21.23)	20(28.17)	18(22.22)	18(22.22)	11(13.92)	16(20.25)	
Somewhat agree	121(30.95)	13(18.31)	29(35.80)	25(30.86)	27(34.18)	27(34.18)	
Strongly agree	67(17.14)	11(15.49)	8(9.88)	15(18.52)	22(27.85)	11(13.92)	
Perceived effectiveness of preventive behavior^5^							
Strongly disagree	9(2.30)	5(7.04)	1(1.23)	1(1.23)	0(0.00)	2(2.53)	*F = 9.00, p = 0.0000*
Somewhat disagree	25(6.39)	4(5.63)	7(8.64)	8(9.88)	3(3.80)	3(3.80)	
Neither agree nor disagree	61(15.60)	21(29.58)	15(18.52)	11(13.58)	8(10.13)	6(7.59)	
Somewhat agree	159(40.66)	28(39.44)	37(45.68)	31(38.27)	39(49.37)	24(30.38)	
Strongly agree	137(35.04)	13(18.31)	21(25.93)	30(37.04)	29(36.71)	44(55.70)	

Most of the variables were reported with frequency and percentage, except for age and capacity, which had mean values and standard deviations.

To assess the effectiveness of different framing strategies on participants’ compliance intentions, ordinal logistic regression (OLR) analyses were performed in both unadjusted and adjusted models. The adjusted models controlled comprehensively for demographic characteristics (gender, age, occupation, education, income level and income changes), health status, cross-regional mobility, family vulnerability to infection, perceived probability of infection, perceived severity of the pandemic, and perceived effectiveness of compliance.

Table 2 reports the ordered-logit coefficients (β) and their 95% confidence intervals for each framing condition relative to the non-framed control group. For ease of interpretation, the discussion in the text converts these coefficients to odds ratios (OR = e^β) and presents the exponentiated confidence limits. The findings in Table 2 demonstrate that all four framing strategies significantly increased participants’ willingness to comply compared to the control group. This provides strong support for Hypothesis 1, which predicted that framed messages (gain or loss) significantly increase individuals’ willingness to comply with epidemic control measures compared to non-framed messages. Specifically, in the adjusted models, participants exposed to the Gain-private frame showed substantially higher willingness to comply (OR = 18.10, 95% CI [7.79, 42.09], *p* < 0.001) relative to the control group. Similar significant increases in willingness were observed for the Loss-private condition (OR = 10.91, 95% CI [4.76, 25.01], *p* < 0.001) and the Gain-social condition (OR = 11.70, 95% CI [4.90, 27.92], *p* < 0.001) when compared to the control. The Loss-social condition exhibited the largest effect size among all frames when compared to the control (OR = 26.03, 95% CI [10.10, 67.06], *p* < 0.001). The robustness of these effects, which persisted even after comprehensive covariate adjustments, reinforces the effectiveness of strategic message framing during epidemic crises. This initial comparison suggests that emphasizing societal losses associated with noncompliance (Loss-social frame) potentially has the strongest motivational influence when juxtaposed with a non-framed message, perhaps due to heightened collective risk awareness and social responsibility during such crises.

**Table 2:** Framing effect on willingness to comply.

	Willingness to comply	
	Unadjusted	Adjusted
Gain-private (vs. non-framed)	2.304^***^ [1.610, 2.997]	2.896^***^ [2.052, 3.739]
Gain-social (vs. non-framed)	2.691^***^ [1.951, 3.430]	2.460^***^ [1.589, 3.329]
Loss-private (vs. non-framed)	2.251^***^ [1.554, 2.948]	2.390^***^ [1.561, 3.219]
Loss-social (vs. non-framed)	3.121^***^ [2.320, 3.921]	3.259^***^ [2.313, 4.206]

^*^p ≤ 0.1; ^**^p ≤ 0.05; ^***^p ≤ 0.01. In the adjusted model gender, age, health status, occupation, education, vulnerable family members, capacity, cross regional, family annual income, income change, perceived probability, perceived severity, and perceived effectiveness were controlled; 95% confidence intervals in brackets.

To examine precisely which framing combination yielded the greatest impact and to test Hypotheses H2, H3, H4a, H4b, H5a and H5b, comparisons of the main framing types were first conducted, along with pairwise comparisons between specific framed conditions, using adjusted ordinal logistic regression as presented in Table 3. These analyses assessed the differences in effectiveness for the general hypotheses and between the specific framed conditions themselves.

**Table 3:** Pairwise comparisons between different frames on willingness to comply.

	Willingness to comply	
	Unadjusted	Adjusted
Gain vs. Loss	−0.081 [−0.540, 0.377]	−0.057 [−0.576, 0.461]
Private vs. Social	−0.907^***^ [−1.382, −0.432]	−0.738^***^ [−1.275, −0.202]
Gain vs. Loss (Private only)	0.356 [−0.260, 0.972]	0.933** [0.126, 1.740]
Gain vs. Loss (Social only)	−0.742* [−1.489, 0.004]	−0.822* [−1.741, 0.096]
Private vs. Social (Gain only)	−0.392 [−1.039, 0.256]	0.059 [−0.749, 0.868]
Private vs. Social (Loss only)	−1.493^***^ [−2.214, −0.771]	−1.800^***^ [−2.700, −0.899]

*p ≤ 0.1; **p ≤ 0.05; ***p ≤ 0.01. In the adjusted model gender, age, health status, occupation, education, vulnerable family members, capacity, cross regional, family annual income, income change, perceived probability, perceived severity, and perceived effectiveness were controlled; 95% confidence intervals in brackets.

Hypothesis 2 predicted that gain-framed messages would be more effective overall than loss-framed messages in promoting preventive compliance, reflecting the expectation that people prefer certain positive outcomes for preventive behaviors. In our experiment, however, this hypothesis was not supported as a general main effect. When averaging across contexts, there was no significant overall difference in compliance between gain-framed versus loss-framed messages (OR = 0.94, 95% CI [0.56, 1.59], *p* = 0.829), indicating that neither framing approach dominated on its own. Instead, the relative effectiveness of gain vs. loss framing depended on the message’s context (as detailed under H4a/H4b below), suggesting that the impact of goal framing is conditional on motivational framing.

Hypothesis 3 posited that social-framed messages would outperform private-framed messages in driving compliance, due to the collective-benefit nature of epidemic control. Our analysis suggest that social framing did elicit higher willingness than private framing (OR = 0.48, 95% CI [0.28, 0.82], *p* < 0.01), thereby supporting our hypothesis 3. However, as detailed under H5a/H5b below, his primary effect was driven by the significant impact of the social-loss framed messages.

We explicitly hypothesized that the impact of gain vs. loss framing would reverse depending on whether the message focused on private or social outcomes. The adjusted pairwise comparisons provided clear evidence for these interaction effects. Within the private-benefit framing context, as predicted by Hypothesis 4a, gain-framed messages were significantly more effective than loss-framed messages in promoting compliance. Participants who received a privately framed message emphasizing personal gains (Gain-private) showed greater willingness to comply than those who received a comparable message emphasizing personal losses (Loss-private). This difference was statistically significant (OR = 2.54, 95% CI [1.13, 5.70], *p* < 0.05), indicating that when focusing on oneself, framing the epidemic measures in terms of gains (e.g., personal safety or benefit) was more compelling than focusing on personal losses. Thus, H4a was supported: gain framing is more persuasive than loss framing for messages centered on private benefits.

In contrast, within the social-benefit framing context, we observed the opposite pattern consistent with Hypothesis 4b: loss-framed messages were more effective than gain-framed messages when the appeal emphasized outcomes for others or society. Participants exposed to the Loss-social frame (highlighting grave consequences for the community if people do not comply) reported higher willingness to comply than those exposed to the Gain-social frame (highlighting benefits to the community if people do comply). This difference was somewhat smaller in magnitude and reached marginal significance (OR = 0.44, 95% CI [0.18, 1.10], *p* = 0.079). Although modest, the trend supports H4b: within a social context, focusing on potential losses to others is more motivating than focusing on potential gains for others. Behaviorally, this result suggests that when people consider collective outcomes, they respond more to warnings of harm (perhaps due to a sense of alarm and responsibility for preventing others’ suffering) than to promises of benefit. This aligns with the notion that negative framing can heighten perceived severity and urgency, especially regarding communal risks. Even though the gain-social message conveyed positive societal outcomes (which might normally be appealing), it was slightly less compelling here—likely because the loss-social message taps into both loss aversion and social responsibility, creating a stronger emotional impetus to act. In sum, H4b was supported at a trend level, indicating that loss framing holds a relative advantage over gain framing in the social domain, in line with our theoretical expectations about heightened sensitivity to societal losses.

H5a examined whether, within a positively framed message (gain frame), emphasizing social benefits yields higher compliance than emphasizing private benefits. H5a was not supported—there was no significant difference between social vs. private framing in the gain context. When messages were gain-framed, it did not matter whether they touted personal gains or social gains: compliance willingness remained equally high in both cases. Statistically, the Gain-Social vs. Gain-Private comparison showed a negligible adjusted difference (OR = 1.06, 95% CI [0.47, 2.38], *p* = 0.886), confirming that participants responded to the gain frame similarly regardless of the beneficiary emphasized. We also considered that in a gain context, people might interpret community benefits as indirectly benefiting themselves too (a “rising tide lifts all boats” perception), thereby blurring differences between social and private appeals.

H5b focused on the flip side of H5a, asking whether within a negatively framed message (loss frame), emphasizing social outcomes is more effective than emphasizing private outcomes. We expected a strong advantage for the social-loss approach, based on both loss aversion and the power of social responsibility. When the message is framed as averting losses, directing attention to societal losses should trigger not only personal loss aversion but also feelings of guilt, empathy, and obligation—a potent motivational mix. People may be more willing to act to prevent harm to others than to prevent harm only to themselves, especially in a pandemic context where one’s behavior directly affects vulnerable people. The result shows that H5b was decisively supported: under loss framing, social-focused messaging was far more effective than private-focused messaging, with the largest observed difference among all pairwise comparisons. The adjusted analysis shows a large contrast (OR = 0.17, 95% CI [0.07, 0.41], *p* < 0.001), indicating that participants hearing about potential societal losses were significantly more willing to comply than those hearing about personal losses. In our study, the combination of a loss frame with a communal target tapped into “greater-than-self” motivations, suggesting that potential losses to the wider society loom psychologically larger than equivalent losses to oneself. This echoes the notion that people may tolerate personal risks to some degree, but the prospect of harming others creates a powerful moral impetus to comply. Notably, among all framed conditions tested, the social-loss frame not only had the highest impact relative to control (Table 2) but also proved significantly superior to its private-loss counterpart (Table 3) in direct comparison. This underscores that highlighting societal losses in a public health crisis is a particularly effective motivator, likely because it raises collective risk awareness and taps into prosocial instincts to protect others.

## 5 Discussion and conclusion

This study investigates the impact of strategic message framing on willingness to comply with health guidance during public health emergencies, addressing a critical challenge in epidemic policy implementation. While prior research extensively explored framing effects in general health behaviors (15, 17), this study uniquely integrates goal-framing (gain vs. loss) and motivational framing (private vs. social) dimensions within the specific and consequential context of epidemic control measures. By doing so, the findings provide nuanced insights for improving policy compliance through tailored public health communications.

Empirically, the study confirms that all tested framing approaches significantly increased compliance intentions compared to no message. However, nuanced differences emerged clearly upon closer examination of how motivational framing (private vs. social) interacted with goal framing (gain vs. loss). While Hypothesis H3 predicted a general stronger impact for social-framed messages, the results of pairwise comparisons showed this effect was conditional, not uniform irrespective of the goal frame. Specifically, when comparing private and social frames in a gain context, there was no significant difference between Gain-Private and Gain-Social frames. This indicates that appealing to social benefits did not significantly enhance compliance over appealing to private benefits when both were framed as gains. In stark contrast, when comparing private and social frames in a loss context, the Loss-Social frame was significantly and substantially more effective than the Loss-Private frame. This highlights that emphasizing potential losses to society is a particularly powerful motivator compared to emphasizing potential losses to oneself. Therefore, the findings demonstrate that the predicted superiority of social framing in H3 is most pronounced and statistically significant when messages are framed in terms of losses, underscoring the importance of considering the interaction between motivational and goal framing. These results offer crucial guidance for strategically leveraging loss-oriented framing in collective contexts.

Moreover, among the tested framing combinations, the loss-social frame exhibited the strongest overall effectiveness in motivating compliance, underscoring a heightened responsiveness to societal losses during public health emergencies. A possible explanation for this robust effect lies in heightened collective consciousness and amplified risk sensitivity triggered by epidemic contexts. Under these high-risk scenarios, emphasizing societal consequences may resonate strongly, prompting individuals to prioritize collective welfare through increased adherence to recommended behaviors. This interpretation aligns closely with prospect theory, suggesting that losses loom psychologically larger than equivalent gains, particularly when decisions affect broader social outcomes (16).

The study’s theoretical contributions are substantial. First, by explicitly integrating both motivational (private vs. social) and goal-framing (gain vs. loss) dimensions, the research enriches existing framing literature, providing a more comprehensive conceptualization of decision-making under conditions of uncertainty and collective responsibility. In particular, this research extends prospect theory beyond individual-level decision contexts to social contexts, revealing complex interactions that clarify when loss-framing may override typical preferences for gain-framed prevention messages.

Second, the findings deepen understanding within dual-process decision-making frameworks (14), highlighting that under crisis conditions, rapid, intuitive System 1 processes—triggered by emotional appeals and immediate social risks—can effectively drive compliance behavior. This extends behavioral science insights into high-stakes public health emergencies, emphasizing the necessity of communication strategies aligned with intuitive, automatic cognitive processes rather than solely relying on rational deliberation (System 2).

Third, the demonstrated superiority of social loss-framing underlines the central role of prosocial motivations and collective responsibility during epidemic scenarios. This insight aligns with broader research emphasizing the importance of collective action frameworks, prosocial identity, and communal responsibility in shaping individual behavior during emergencies (29, 30). By explicitly situating compliance behavior within a collective context, the study identifies communication strategies that effectively tap into shared social identities and collective responsibility to enhance policy adherence.

Practically, these results offer actionable insights for policymakers and public health authorities. The clear effectiveness of loss-social frames suggests that public health messaging during crises should explicitly emphasize societal risks, clearly articulating how noncompliance exacerbates broader social harm. Messages focusing on the communal consequences of failing to adhere to health guidelines—such as overwhelming healthcare systems or increasing infections among vulnerable populations—could be quickly and widely disseminated through mass media campaigns, digital channels, and public service announcements. Additionally, framing interventions are notably cost-effective, scalable, and readily adaptable, making them particularly suitable for rapid deployment in resource-limited or emergency contexts.

Nevertheless, several limitations provide critical avenues for future research. A significant consideration is the potential for “good participant bias” or demand characteristics in the experimental conditions. This bias suggests that participants, particularly in the framed conditions, might have inferred the study’s hypothesis and adjusted their self-reported willingness to comply to align with the expected behavior, anticipating that they “should” follow the guidance supported by the frames, unlike those in the control condition where such an expectation might be less pronounced. While our findings consistently showed that framed messages significantly increased compliance intentions compared to the control condition, with effects persisting even after comprehensive covariate adjustments, reinforcing their robustness, it is inherently difficult to definitively separate this potential effect from the direct results due to framing.

This potential for bias is primarily related to our first limitation: the reliance on behavioral intentions rather than actual compliance behaviors. Although behavioral intentions are recognized as strong predictors of actual behaviors, the literature consistently documents discrepancies between what people intend to do and what they actually do (31). In the context of our study, this means participants might report a higher willingness to comply due to social desirability or a desire to be perceived as a “good participant” aligning with the framed message, even if their actual behavior in a real-world scenario might differ.

Furthermore, a methodological limitation that could potentially exacerbate this bias is the reliance on single-item measures for key variables, including willingness to comply, perceived probability of infection, perceived severity, and perceived effectiveness of compliance. While the measure for willingness to comply was adapted from previous validated research, using a single question to capture complex psychological constructs may limit the reliability and comprehensive capture of these variables compared to multi-item scales. Such single, direct questions might be more susceptible to participants reporting what they believe is the desired answer, rather than their nuanced or complex internal state. To mitigate these concerns and provide more robust validation of framing effects, future studies should incorporate observational or longitudinal designs to measure actual compliance behaviors such as mask usage, adherence to social distancing guidelines, or vaccination uptake. Additionally, employing validated multi-item scales would enhance the reliability and validity of the measurements, thereby strengthening the confidence in the observed framing effects.

Second, although randomized experimental conditions effectively controlled for potential confounders, the relatively moderate sample size (*N* = 391) limits generalizability and statistical power, particularly when detecting subtler effects. Additionally, the sample was predominantly female (68.8%), which further limits the generalizability of the findings to populations with different gender distributions. Replications with larger, more diverse samples across different demographic and cultural groups, including a more balanced gender representation, would strengthen external validity and generalizability, offering robust insights applicable across broader contexts and allowing for the exploration of potential gender-specific responses to framing strategies.

Third, it is important to acknowledge the specific context of this study: a simulated hypothetical resurgence scenario of COVID-19 following an initial wave. While this scenario was chosen for its ecological validity, mirroring actual public health emergencies by imagining a new, highly hazardous variant and re-implemented non-pharmaceutical interventions, it raises crucial questions about the direct applicability of these findings to a completely novel pandemic or the very early stages of an initial outbreak. Public attitudes, perceptions of risk, and compliance behaviors may dynamically evolve across epidemic phases. Future longitudinal studies could explore how framing effectiveness varies at different stages of an epidemic, potentially guiding dynamic message adjustments throughout a public health crisis. For instance, loss-framing might dominate early high-risk stages, whereas gain-framing could become effective during recovery or vaccination campaigns.

Finally, the cultural context of the study—conducted in China, a society emphasizing collective identity and social responsibility—might significantly influence framing effectiveness. Comparative studies in culturally individualistic societies could examine how cultural values moderate framing effects. Additionally, psychological moderators such as trust in government, risk tolerance, and individualistic versus collectivistic orientation should be systematically explored to further refine culturally tailored framing interventions.

Indeed, prior research emphasizes the significance of these cultural and psychological moderators in shaping compliance behaviors during health crises. For instance, Han et al. demonstrated that greater trust in government positively influenced preventive behaviors and prosocial actions throughout the COVID-19 pandemic (32). Similarly, Lim et al. found that increased trust in governmental messaging enhanced adherence to recommended health measures in Singapore (33). Conversely, Schmelz argued that stringent enforcement mechanisms may unintentionally undermine voluntary compliance, particularly in contexts where trust in government institutions is relatively low (34). Additionally, recent work by Stanulewicz highlighted how individuals’ threat appraisal and source credibility significantly moderate the persuasiveness of public health messages (35). These insights suggest a clear need to account for such moderators in future framing studies, facilitating more effective culturally and psychologically tailored interventions.

In conclusion, this study provides important theoretical and practical contributions, demonstrating how targeted message framing can enhance compliance with public health measures during epidemic emergencies. While the findings offer valuable insights, it is essential that the results be interpreted with caution, as they are based on a single study conducted in one particular context. Specifically, the data were collected within a specific time window between epidemic waves of the COVID-19 pandemic, which may influence public perceptions and behaviors, and in China, a cultural setting that may emphasize collective identity and social responsibility, potentially affecting framing effectiveness. The study highlights particularly powerful effects of loss-social frames, emphasizing societal consequences of noncompliance, and integrates behavioral economic insights, prospect theory, and dual-system cognitive frameworks to advance theoretical understanding and provide actionable insights for policy communications. As global public health threats persist, strategically informed framing approaches offer vital tools for improving voluntary public adherence; however, their effectiveness warrants further validation across diverse contexts and populations to strengthen external validity and generalizability.

## Data Availability

The raw data supporting the conclusions of this article will be made available by the authors, without undue reservation.
