# Supplementary material for: Nudging epidemic policy compliance: experimental insights into message framing
Source: Front Public Health. 2025 Jul 23;13:1587987. doi: 10.3389/fpubh.2025.1587987 (PMC12325323; doi:10.3389/fpubh.2025.1587987)
Supplement: Supplementary file 1 [file Table_1.docx]

Appendix Table A. Message Prompts and Options for Experimental Conditions Translate into English.

| Message prompt | Imagine yourself in the early stages of a sudden, highly hazardous infectious disease outbreak, which spreads rapidly and has a severe illness burden with a high mortality rate. Representative past outbreaks include Ebola virus, SARS, swine flu, and COVID-19. The infectious disease currently lacks an effective vaccine and specific treatment. In order to control the spread of the epidemic, relevant authorities have formulated and advocated the following public health policies for all members of society to adhere to:   1. Restricting movement of individuals: Comply with national and local epidemic prevention and control measures and regulations. Actively participate in quarantine work as required, and cooperate with measures such as population flow adjustment, travel restrictions, and area lockdowns. Avoid travel and gatherings unless absolutely necessary, and do not enter epidemic-stricken areas without permission. 2. Strengthening personal protection: Wear masks properly when going out, practice good personal hygiene, maintain social distancing, and cover your mouth and nose when coughing or sneezing. Maintain good hygiene habits, enhance ventilation when at home, ensure cleanliness and disinfection of the household, and pay attention to personal hygiene. Wash hands frequently and maintain good hand hygiene. 3. Conducting health monitoring: Proactively monitor your own and your family's health conditions. If symptoms occur, seek medical attention promptly and report immediately without concealing the illness. Stay informed about the epidemic information released by authoritative channels, maintain psychological well-being and cope effectively. Refrain from believing or spreading rumours to avoid unnecessary panic and negative impact. | | |
| --- | --- | --- | --- |
| Private | Gain | Gain-framed (Experiment1) | If you comply with these epidemic policies, it can bring the following benefits to you:  Reduce your risk of getting infected  help you maintain good health and avoid illness  ensure that your daily life  income remain stable and unaffected. |
|  | Loss | Loss-framed (Experiment2) | If you do not comply with these epidemic policies, it can bring the following risks to you:  You may become ill  Experience severe symptoms or even face life-threatening situations  Disrupt your normal life  Disrupt economic stability. |
| Social | Gain | Gain-framed (Experiment3) | If you comply with these epidemic policies, it can bring the following benefits to society:  Reducing the risk of disease transmission  Protecting vulnerable populations (such as the elderly and children)  Easing the pressure on healthcare systems  Accelerating the recovery of social order and the economy. |
|  | Loss | Loss-framed (Experiment4) | If you do not comply with these epidemic policies, it can bring the following risks to society:  Increase the risk of infecting others (especially vulnerable individuals)  Worsen the spread of the epidemic  Add to the healthcare system’s burden  Delay both social recovery and economic stability. |
| *Are you willing to comply with these epidemic policies?* | | | |
